# Application of ferroelectric materials for improving output power of energy harvesters

**DOI:** 10.1186/s40580-018-0163-0

**Published:** 2018-11-02

**Authors:** Tae Yun Kim, Sung Kyun Kim, Sang-Woo Kim

**Affiliations:** 1 School of Advanced Materials Science and Engineering, Sungkyunkwan University (SKKU), Suwon, 16419 Republic of Korea; 2 Department of Materials Science & Metallurgy, University of Cambridge, Cambridge, CB3 0FS UK

**Keywords:** Ferroelectricity, Energy harvesting, Piezoelectricity, Triboelectric effect, Photovoltaic effect

## Abstract

In terms of advances in technology, especially electronic devices for human use, there are needs for miniaturization, low power, and flexibility. However, there are problems that can be caused by these changes in terms of battery life and size. In order to compensate for these problems, research on energy harvesting using environmental energy (mechanical energy, thermal energy, solar energy etc.) has attracted attention. Ferroelectric materials which have switchable dipole moment are promising for energy harvesting fields because of its special properties such as strong dipole moment, piezoelectricity, pyroelectricity. The strong dipole moment in ferroelectric materials can increase internal potential and output power of energy harvesters. In this review, we will provide an overview of the recent research on various energy harvesting fields using ferroelectrics. A brief introduction to energy harvesting and the properties of the ferroelectric material are described, and applications to energy harvesters to improve output power are described as well.

## Introduction

Two of the most important trends in recent electronic technology have been the size reduction and functional improvement of mobile electronic devices. Mobile electronic devices are small, portable, and contain a variety of information that is instantly accessible at all times, including the ability to share and communicate information wirelessly. These devices are becoming even smaller and lighter so that they can be wearable or attachable to objects that can be used daily, such as a watch, glasses, or clothes. All devices that are based on microelectronic technology require a lot of external power supply due to their increased functions, and batteries are the most important power source for mobile electronic devices. However, batteries take up increasingly significant parts of the overall device volume and weight as the electronic devices are miniaturized. Moreover, battery technology is limited in energy capacity per volume for supplying sufficient energy to a mobile electronic device.

Therefore, many studies have been focused on reducing power consumption and designing energy efficient devices to reduce the sizes but extend the lifetimes of the batteries. Nevertheless, the batteries must be replaced or recharged after being discharged, and this is an obstacle to realizing always-on wearable electronic devices. In order to make up for this problem, we need to develop an energy harvesting system that can harvest and reuse energy sources from the ambient environment. Energy harvesters convert various environmental energy sources such as mechanical stress, vibration, light, and heat, etc. to electrical energy. Each energy source can be converted to electrical energy by each coupled-physical phenomenon such as piezoelectric, triboelectric photovoltaic, and thermoelectric (or pyroelectric) effects. The amount of output energy obtained from piezoelectric effect is ~ 5.92 μW/cm^2^ [1], triboelectric effect is ~ 0.7 mW/cm^2^ [2], photovoltaic effect is ~ 22.1 mW/cm^2^ [3], and pyroelectric effect is 1.4 μW/cm^2^ [4]. The working principle of each energy harvester is different, but basically, electric current is generated by internal polarization or potential. Therefore, increasing the polarization density is important for improving output power of energy harvester. Conventional materials have limitation in increasing internal polarization because of low polarization density. Moreover hardness of the conventional materials hinders application to wearable devices. However introducing novel materials with strong and permanent polarization, ferroelectric materials, can overcome these limitations. In this review article, ferroelectric materials in energy harvesters are addressed. Ferroelectric materials have permanent dipole moments once electric field is applied, so polarization density can be increased through the insertion of ferroelectric materials. First, we will briefly describe the types of ferroelectric materials as well as the basic theory of energy harvesting technologies. Then, recent applications of ferroelectric materials in energy harvesting devices are discussed.

## Ferroelectric materials

**Fig. 1:**
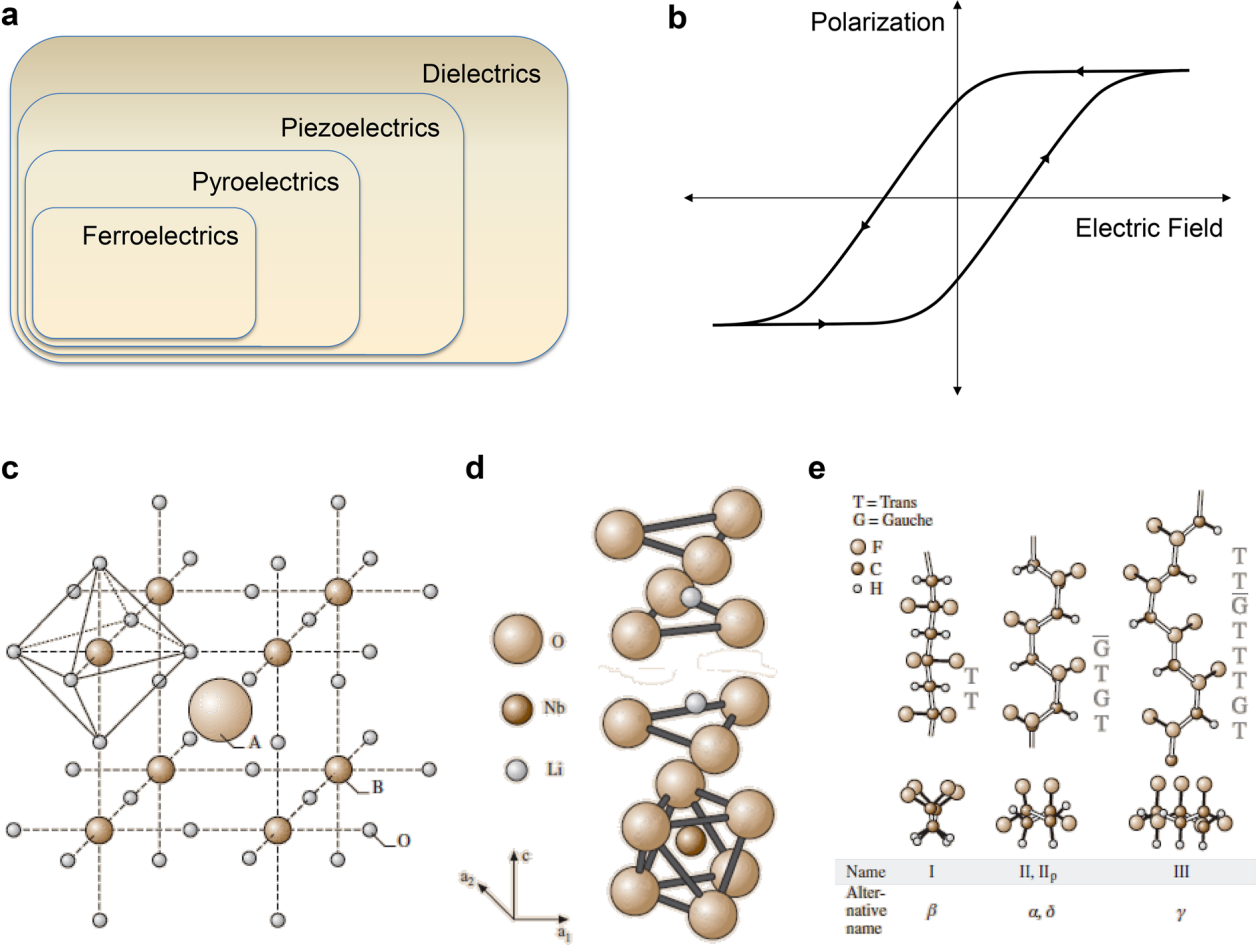
**a** Ferroelectrics in Venn diagram of dielectric classes. **b** Hysteresis loop of ferroelectric material in P–E curve. **c** Crystal structure of perovskite **d** ilmenite, and **e** polymeric ferroelectric material (Reproduced from [5] with copyright permission from 2006 Springer)

Ferroelectric materials can be defined as dielectric materials in which polarization remains permanently, even after removing the applied electric field. Moreover, the direction of the dipole moment can be switched by applying electric field. Among the 32 crystal classes, 21 have non-centrosymmetric and 20 have direct piezoelectricity among them, which forms polarization through mechanical stress. Ten of the piezoelectric crystal classes have spontaneous electric polarization and this electric polarization varies with temperature change which is called pyroelectricity. Some of the pyroelectric materials are ferroelectric materials whose polarization can be reversed by external electric field. Therefore, ferroelectric materials have both pyroelectricity and piezoelectricity. Their relationships are illustrated in a Venn diagram in Fig. 1a [5].

The ferroelectricity can be tested by measuring polarization as a function of electric field. Ferroelectric materials have spontaneous polarization, and this varies with external electric field, so in a polarization versus electric field curve, a hysteresis loop is shown (Fig. 1b). However, the ferroelectricity is shown only after the phase transition below a certain temperature, called Curie temperature (T_C_). Above the Curie temperature, ferroelectricity disappears and paraelectricity is shown.

### Perovskite ferroelectric materials

These materials have perovskite structures, like BaTiO_3_, whose general chemical formula is ABO_3_, where A and B atoms are cations. Normally the A cation has radius of 1.2–1.6 Å and B cation has one of 0.6–0.7 Å. A atoms are positioned at the cube corner and oxygen atoms are positioned at the face center and form an octahedron surrounding the B atom which is positioned at the body center, as graphically illustrated in Fig. 1c [5]. Under electric field, the position of B cation shifts, then the geometrically unbalanced electrical charge forms a dipole moment.

### Ilmenite ferroelectric materials

The ilmenite ferroelectric materials have the same chemical formula as perovskite materials, ABO_3_, e.g. LiNbO_3_ and LiTaO_3_. However, the A cation is too small to fill the position of the perovskite crystal coordinate [6, 7]. Oxygen atoms comprise hexagonal close-packed layer, and A and B atoms are positioned at the octahedron site between layers (Fig. 1d) [5].

### Polymeric ferroelectric materials

The first discovered and the most representative polymeric ferroelectric material is polyvinylidene fluoride (PVDF) [8–10]. Polymers have long carbon backbone, so their structure is complex and has a lot of configurations depending on whether the neighboring carbon bonds are trans or gauche. Among the configurations of PVDF described in Fig. 1e [5], the β-phase has all trans configuration. The fluorine atoms have the strongest electronegativity, resulting C–F polar bond so that PVDF molecule has dipole moment in perpendicular direction to its carbon chain. However, the dipole moments of the pristine polymer chains are not arranged in single direction, so the net polarization is zero. Therefore, a strong electric field is required to arrange the dipole moments of chains, which is called electrical poling. In addition, copolymer with trifluoroethylene (10–46%) helps the formation of β-phase.

## Ferroelectric materials in energy harvesting

Energy harvesting utilizes various energy sources, including mechanical, thermal, and solar energies. Each energy source can be converted to electrical energy through each coupled-physical phenomenon, but basic principle is same: the variation of the internal dipole moment or potential. Therefore, the introduction of ferroelectric materials to energy harvesters can increase dipole moments and potential in the devices due to the strong polarization in the ferroelectric materials so that conversion efficiency can be enhanced.

### Piezoelectric energy harvesting

#### Piezoelectric effect

Piezoelectric effect is a coupling phenomenon of mechanical strain and electric charge separation. When mechanical stress is applied to the materials which have asymmetric crystal structures, the crystal structure is deformed, resulting in a separation of the center of charges. The charge separation induces a dipole moment that is proportional to stress or strain (direct piezoelectric effect). Since this was first discovered by Pierre Curie and Jacques Curie in 1880 using quartz and Rochell salt [11, 12], many piezoelectric materials, such as PbZr_0.52_Ti_0.48_O_3_ (PZT), BaTiO_3_ (BTO), ZnO, and PVDF have been studied and had their piezoelectric constants measured. The induced dipole moment by piezoelectric effect, piezoelectric dipole moment (*P*_piezo_), is described by the linear equation:

1 $$ P_{\text{piezo}} = {\text{d}} \cdot T = {\text{d}} \cdot {\text{Y}} \cdot S = {\text{e}} \cdot S $$

where d (C/N or m/V) and e (C/m^2^) are piezoelectric coefficients, *T* (N/m^2^) is stress, Y (N/m^2^) is Young’s modulus, and *S* is strain.

Due to the coupling effect of mechanical strain and electric charge separation in piezoelectric effect, the piezoelectric effect has been exploited to convert mechanical energy. Energy harvesting using the piezoelectric effect was first introduced by Wang’s group using piezoelectric semiconducting ZnO nanowires in 2006 [13]. Since then, a lot of research on piezoelectric energy harvesters, called piezoelectric nanogenerators (PENG), has been reported. Many researchers have attempted to enhance the output performance of PENG by designing new devices. Among the various factors to increase output performance, the development of a material with a high piezoelectric coefficient is the most important.

Ferroelectric materials have piezoelectricity as well, and their piezoelectric coefficient is relatively high (d_33_ of PMN-PT ferroelectric ceramic is 630 pC/N [14]). Initially, dipole moments in ferroelectric material are randomly aligned so it has neither polarization nor piezoelectricity. However, once strong electric field is applied, dipole moments are aligned in a single direction and piezoelectricity is formed. Therefore, PENGs made of ferroelectric materials have been reported.

#### Thin film perovskite PENG

**Fig. 2:**
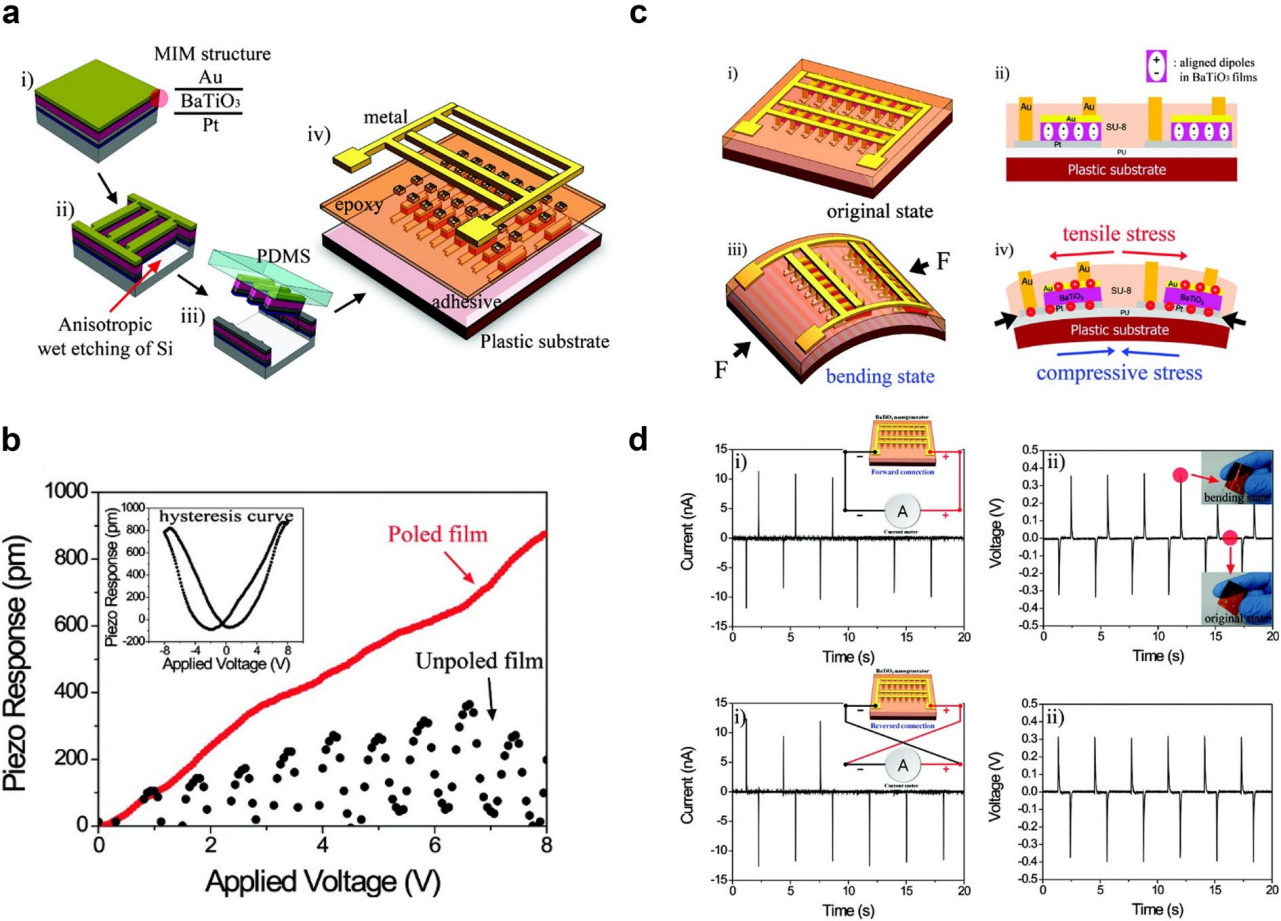
**a** Fabrication process of MIM structure and schematic structure of PENG with BTO thin film. **b** PFM measurement of BTO thin film prior to and after poling. **c** Deformation of BTO thin film under mechanical stress and charge induction. **d** Output current and voltage of PENG with fore and reverse connection (Reproduced from [15] with copyright permission from 2010 American Chemical Society)

Ferroelectric ceramics with perovskite structure have relatively high piezoelectric coefficients, so PENGs made of piezoelectric ceramics, such as BTO [15, 16], PZT [17–19], ZnSnO_3_ [20], and Pb(Mg_1/3_Nb_2/3_)O_3_-xPbTiO_3_ (PMN-PT), show high output powers [21–23]. However, ceramic is a rigid material so it should be deposited as a thin film in order to be flexible. Park et al. [15] reported a thin film BTO based PENG in 2010. The MIM (metal–insulator-metal, Au/BTO/Pt) structure was patterned with a ribbon structure (300 μm × 50 μm) array by photolithography and gas-based ICP-RIE etching. The patterned MIM structure was transferred onto a plastic substrate (Kapton film) using a polydimethylsiloxane (PDMS) stamp. Finally, SU8 epoxy was spin-coated on MIM structure and a metal grid was connected to the top and bottom electrodes. Schematic fabrication process and device image are illustrated in Fig. 2a.

After fabrication, BTO film was poled with an electric field of 100 kV/cm for 15 h at 140 °C in order to align ferroelectric polarization and enhance the piezoelectric output. The piezoelectric coefficient (d_33_) was characterized using piezoresponse force microscopy (PFM) and compared the value prior to and after poling. By the poling process with external electric field, ferroelectric polarization becomes stronger. As shown in Fig. 2b, before poling the measured d_33_ is 40 pm/V but after poling it increased up to 105 pm/V which fits with the previous report (d_33_ = 30–100 pm/V [24, 25]). Besides, hysteresis loop of piezo response in the inset clearly shows ferroelectric behavior of BTO after poling.

The PENG with BTO thin film on flexible substrate is driven by compressive force and bending. Then, tensile stress is applied to BTO film. The deformation of the BTO film by tensile stress generates piezoelectric polarization and induces charge induction in electrode resulting in electrical current (Fig. 2c). Figure 2d shows the output current (~ 10 nA) and voltage (0.3 V) of flexible BTO PENG with 1350 MIM structure arrays by periodic bending and unbending. The BTO based PENG shows the possibility of ferroelectric ceramic material for flexible and high output energy harvesters through thin film deposition and the transferring technique.

#### Ferroelectric-polymer composite PENG

In order to further enhance output power, other ferroelectric materials with higher piezoelectric coefficients such as PZT and PMN-PT were used for energy harvesters [1, 18, 21, 22]. The high-power PENGs with thin film ferroelectric materials were integrated on flexible plastic substrate and utilized for bio-implantable devices [19, 21]. Although the output power of PENGs successfully increased up to an open-circuit voltage of 200 V and short-circuit current density of 150 μA/cm^2^ [18], ferroelectric materials are deposited as thin film, resulting in the limitation of output power and fabricating large area devices. Moreover, rigid ferroelectric thin film cannot be used under strong force.

**Fig. 3:**
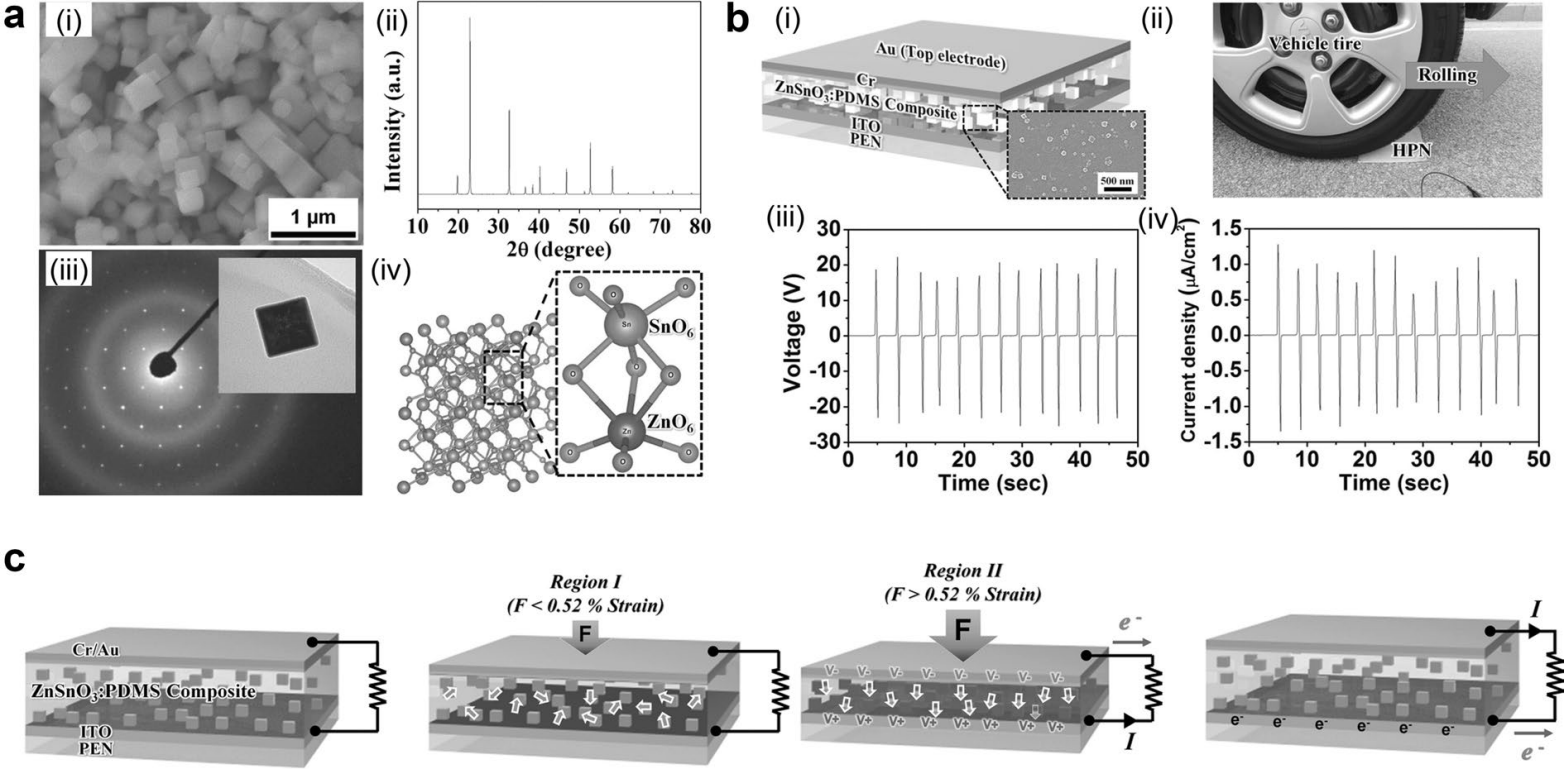
**a** SEM image of ZnSnO_3_ nanocubes and crystal structure characterization using XRD and SAED pattern. **b** Schematic structure of composite based PENG and output performance by vertical stress using vehicle. **c** Working mechanism of charge induction by stress-induced poling effect (Reproduced from [20] with copyright permission from 2014 John Wiley & Sons)

In order to solve these problems, polymer supported ferroelectric powders in PENG have been reported [16, 20, 26]. Polymer matrix-ferroelectric powder composite has advantages in large area fabrication due to easy fabrication process and low-cost, high stress application, and mechanical durability. In particular, ZnSnO_3_ is an ecofriendly and biofriendly lead-free piezoelectric/ferroelectric material and used as a high-power energy harvester without electrical poling [20]. ZbSnO_3_ powder having nanocube morphology with size of 100–200 nm and crystal structure is shown in Fig. 3a. The XRD pattern indicates that the crystal structure of ZnSnO_3_ is rhombohedral. The rhombohedral structure of ZnSnO_3_ is comprised of two octahedral ZnO_6_ and SnO_6_. As illustrated, each octahedron has three long bonds and three short bonds, so Sn and Zn atoms are placed on a deviated position from the octahedron center, resulting in non-centrosymmetry and ferroelectricity.

Well-mixed ZnSnO_3_:PDMS composite was fabricated into PENG and the output power was measured by applying vertical compressive force using vehicle, as described in Fig. 3b. The open-circuit voltage of 20 V and short-circuit current density of 1 μA/cm^2^ were measured. Detailed working mechanism is described in Fig. 3c. At low strain, most of the strain occurs in PDMS matrix so the actual strain in nanocubes are small. As a result, randomly aligned minor piezoelectric potential is induced. However, at a high strain, enough compressive force is applied to nanocubes, so piezoelectric polarization is generated and aligned in a single direction due to stress-induced poling effect [27–30]. Therefore, this result shows high performance of ferroelectric-polymer composite PENG and its promising application in large area and under high pressure.

#### Polymeric ferroelectric based PENG

The ferroelectric powder-embedded polymer composite shows high output power and mechanical stability but is not acceptable for low magnitude and frequency input force. Poly(vinylidene fluoride) (PVDF) is one of the representative ferroelectric polymers. Its copolymer poly(vinylidenefluoride-co-trifluoroethylene) (PVDF-TrFE) has a high piezoelectric coefficient of d_31_ = 25 pC/N, d_33_ = 40 pC/N [31] and its flexibility is promising for application in fully flexible, foldable, twistable, and stretchable PENG [32]. Previously reported PENGs comprised of plastic substrate and metal electrode have limitation in flexibility and stretchability. However, the semi-metallic two-dimensional carbon material, graphene, is a promising electrode for flexible electrode due to its high mechanical durability and elasticity (1 TPa) [33].

**Fig. 4:**
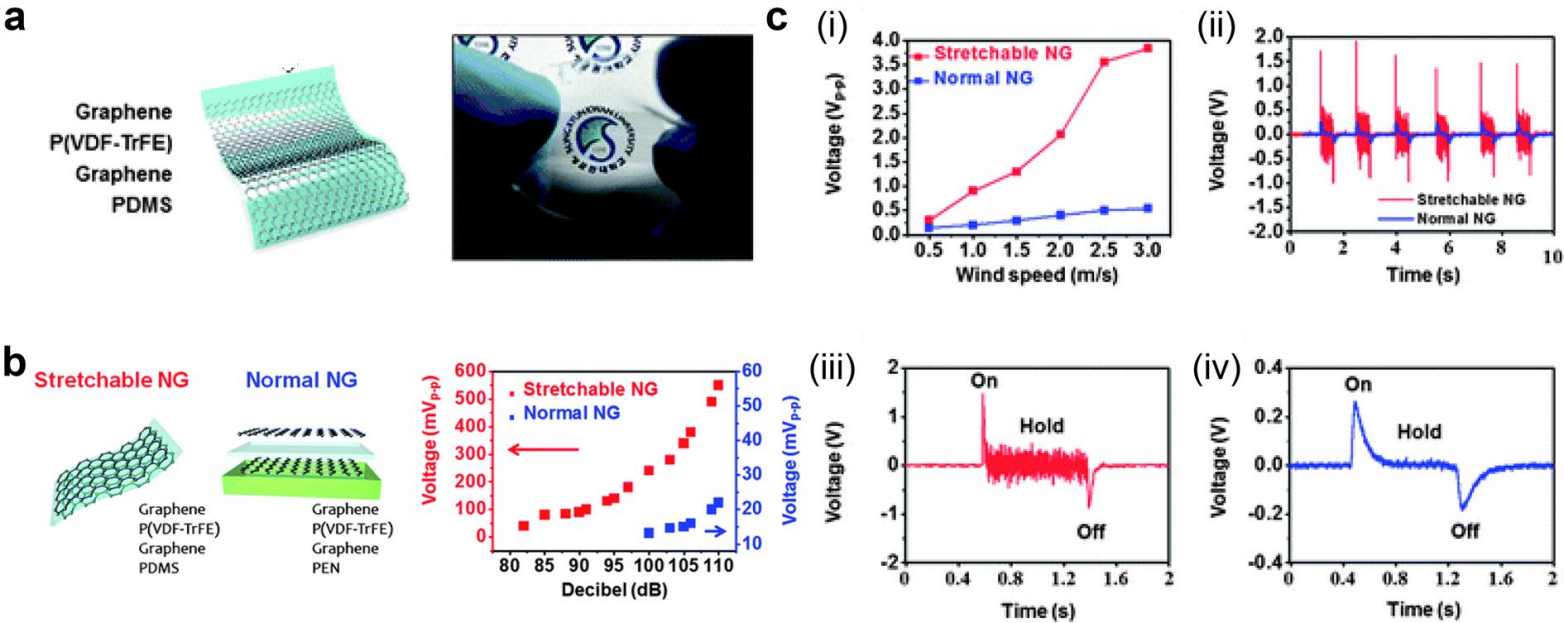
**a** Schematic structure and real image of polymeric ferroelectric material based PENG. **b** Output voltage measurement with applied sound wave, and **c** wind (Reproduced from [32] with copyright permission from 2013 Royal Society of Chemistry)

Lee et al. [32] developed highly sensitive P(VDF-TrFE) PENG, which is comprised of PDMS polymer substrate and P(VDF-TrFE) sandwiched with graphene electrodes (Fig. 4a). The output voltage of the highly sensitive PENG made of P(VDF-TrFE) and graphene electrodes were investigated and compared to PENG with PEN substrate under application of sound wave (82–110 dB at 100 Hz) as shown in Fig. 4b. P(VDF-TrFE) PENG shows enhancement of voltage from 50 mV to 600 mV because of its highly sensitive response to input sound wave. In contrast, the PENG on the PEN substrate has no output signal at low power of sound wave (85–95 dB) but increases from 10 mV to 22 mV at 100–110 dB.

Furthermore, the output voltage was investigated with wind flow (Fig. 4c). With wind speed of 0.5–3 m/s, peak to peak output voltage of P(VDF-TrFE) PENG increased from 0.3 V to 3.9 V while PENG on PEN substrate increased from 0.1 V to 0.5 V. Detailed analyses of single peaks are compared in Fig. 4c(ii). When the continuous wind flows, P(VDF-TrFE) PENG on PDMS substrate flutters because of its flexibility and sensitivity to low magnitude strain so continuous output signals are observed. However, PENG on PEN substrate shows signal only when wind is turned on and off instantly as depicted in Fig. 4c(iii) and (iv). In conclusion, ferroelectric polymer shows very promising result as a high output piezoelectric energy harvester.

### Triboelectric energy harvesting

#### Triboelectric effect

Triboelectric effect is the charge exchange between two materials through contact or rubbing each other. Although the detailed mechanism of triboelectric effect remains elusive, there are four possibilities: electron transfer, ion transfer, material transfer, and mechano-chemistry [34]. Triboelectric charging occurs by complex of these four phenomena. Numerical prediction of triboelectric charging is not possible yet because there are too many factors to determine triboelectric effect, but triboelectric charge polarity is predictable using triboelectric series [34, 35].

Static charges by triboelectric effect have been considered as disturbance to human health and especially industry because the charges have an effect on electric devices. Therefore, there have been efforts to prevent the triboelectric effect. However, prof. Zhong Lin Wang’s group invented a new type of energy harvester called a triboelectric nanogenerator (TENG) which exploits the triboelectric effect in 2012 [36]. TENG extend energy harvesting field more widely due to its simple structure, light weight, and high output power.

Basically, TENG is based on a plane electric field from surface charge by triboelectric effect. Therefore, the electric potential (*V*) of TENG is described by Gauss’s law:

2 $$ V = \frac{{\sigma_{\text{t}} }}{{\upvarepsilon_{0}\upvarepsilon_{\text{r}} }}d $$

where *σ*_t_ is triboelectric charge density, *d* is the gap distance between two plates, and ε_0_ and *ɛ*_r_ are vacuum and relative permittivity respectively. The potential attracts count charges in each electrode when two electrodes are electrically connected by external circuit. The potential is function of gap distance, so output voltage and current are generated according to movement of the plates.

As can be seen in the equation, triboelectric charge density is the most important factor when designing TENG. The triboelectric charge density is determined only by surface property of material. Previously, many researchers have tried to increase the number of fluorine atoms by using Teflon film [37], plasma treatment [38] or self-assembled monolayer (SAM) [39] to enhance output power. The ferroelectric materials have spontaneous polarization so they can enhance the output power of TENG. There have been TENGs supported by ferroelectric polarization.

#### Controllable charge transfer by ferroelectric polarization

In TENG, there is charge transfer between two materials. Generally, the amount and polarity of charge is determined by material properties, especially work function. However, work function is hardly modulated, so controlling triboelectric effect of existing material is very limited. Introduction of ferroelectric material can control and increase triboelectric charging behavior due to its switchable and controllable polarization.

**Fig. 5:**
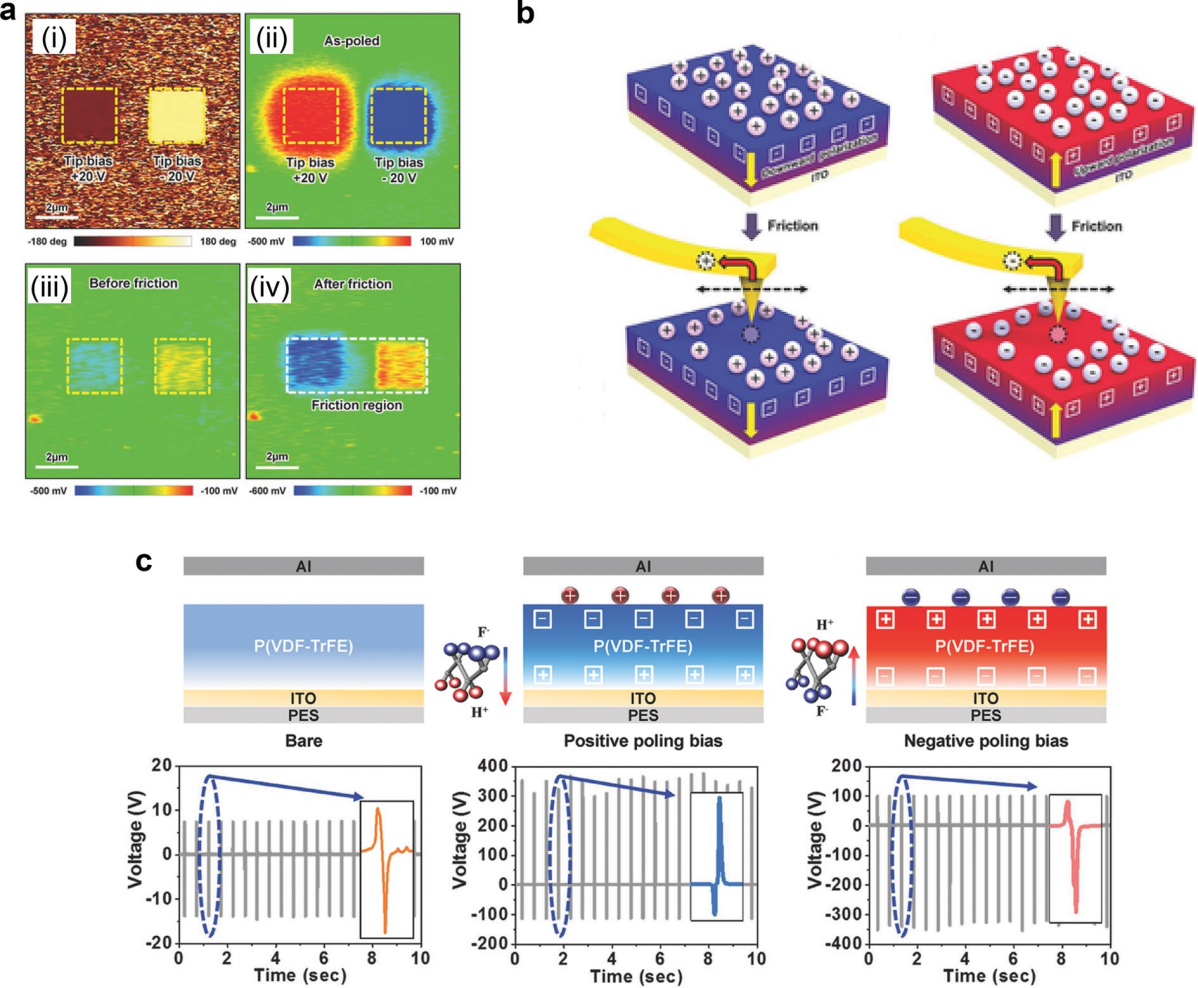
**a** Triboelectric characteristic of P(VDF-TrFE) after poling with opposite bias. **b** Charge transfer from P(VDF-TrFE) surface by rubbing AFM tip. **c** Controlling triboelectric output voltage of P(VDF-TrFE) with different ferroelectric polarizations (Reproduced from [42] with copyright permission from 2016 John Wiley & Sons)

Atomic force microscopy (AFM) is a very good tool for investigating ferroelectric and triboelectric behavior, because both electrical polarizing and characterizing ferroelectric polarization are available [40, 41]. Lee et al. [42] investigated triboelectric behavior of P(VDF-TrFE) polymer affected by triboelectric polarization. First, P(VDF-TrFE) surface was polarized by applying positive and negative bias voltage using AFM tip. The polarization state was characterized by PFM phase measurement and showed different states with different bias, shown in Fig. 5a(i). During the electrical poling process, the charges are over-injected from the AFM tip, so the surface potential image at the initial state in Fig. 5a(ii) shows polarity opposite to ferroelectric polarization. However, the over-injected charges are discharged as time goes on and surface potential shows ferroelectric polarization in Fig. 5a(iii). After surface potential become stable, the P(VDF-TrFE) surface was rubbed with AFM tip to investigate triboelectric effect. The surface potential on the region which has each different direction of ferroelectric polarization became enhanced after rubbing (Fig. 5a(iv)). The charge transfer process by rubbing AFM tip is illustrated in Fig. 5b schematically. Even at a stable state, there are screen charges on the polarized region, and these charges are transferred to the AFM tip during the rubbing process.

The triboelectric behavior affected by ferroelectric polarization was also investigated in an energy harvesting device (TENG). Following the fabrication of TENGs with P(VDF-TrFE) film, each devices was positively or negatively polarized as shown in Fig. 5c, and periodic force was applied to TENGs in order to obtain triboelectric output voltage. The P(VDF-TrFE) films with different applied bias voltage have different direction of polarization so output voltage directions different as well. Moreover, both positively and negatively polarized P(VDF-TrFE) film shows high output voltage than bare P(VDF-TrFE) film because of well-oriented polarization. Conventionally triboelectric property is fixed and determined by tribo-series, but this result shows it can be modified in ferroelectric materials.

#### Ultrahigh triboelectric charge density in TENG by ferroelectric layer

In order to increase the output power of TENG, researchers have tried post treatments such as ionized-air injection [38], self-assembled monolayer [39]. It was found that surface charge density of 240 μC/m^2^ can be obtained by ionized-air injection but long-term stability is not secured. Ferroelectric materials have permanent polarization, so it is expected that the output performance can be enhanced through the use of ferroelectric materials with long-term stability.

**Fig. 6:**
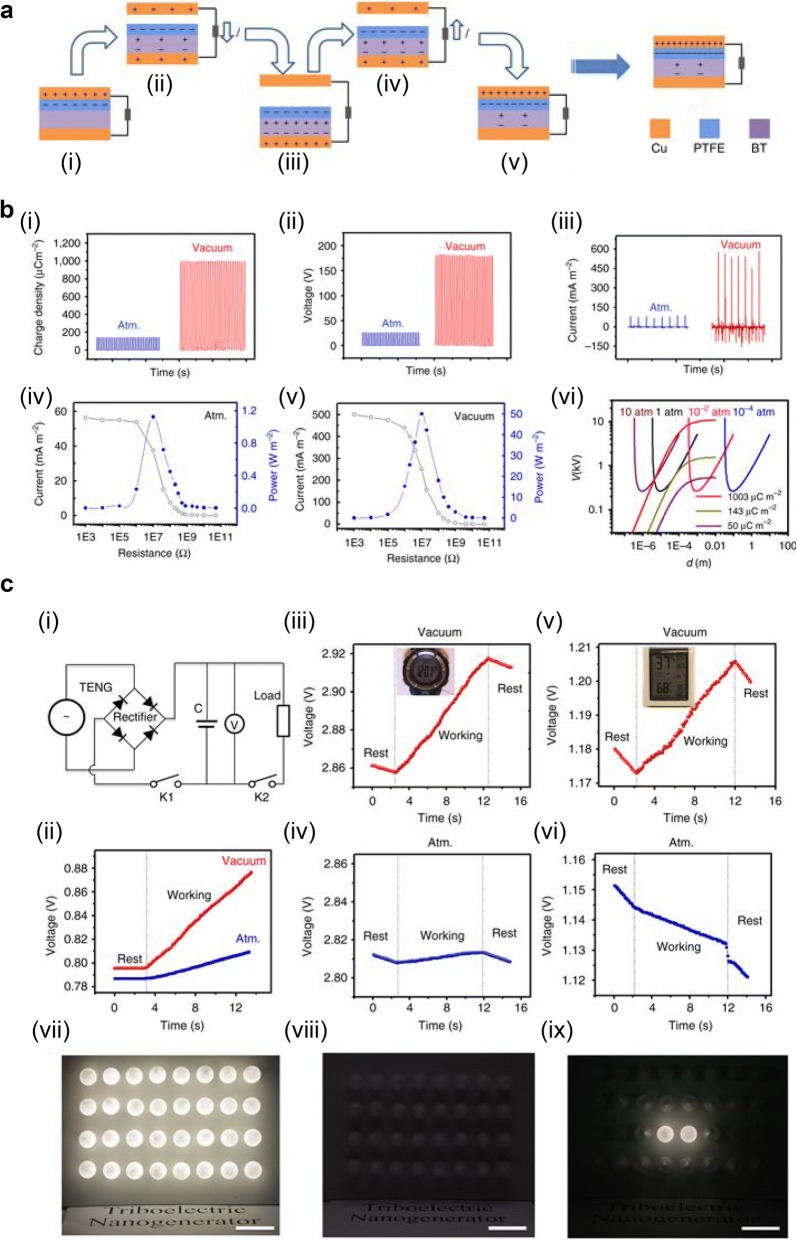
**a** Schematic structure and working sequence of TENG with ferroelectric polarization. **b** Output performance of TENG in atmosphere and high vacuum. **c** Charging supercapacitor while working electronics simultaneously, and powering LEDs (Reproduced from [43] with copyright permission from 2017 Nature Publishing Group)

Wang et al. [43] introduced a ferroelectric layer in TENG in order to increase surface charge density. Figure 6a shows the schematic structure of TENG consisting of Cu top and bottom electrodes, and polytetrafluoroethylene (PTFE) layer on the barium titanate (BT) ferroelectric ceramic. Upon initial contact (Fig. 6a(i)), positive and negative triboelectric charges are generated on the top Cu and PTFE surfaces, respectively. When top Cu and PTFE become separated (Fig. 6a(ii)), ferroelectric polarization is induced in the BT layer by electric potential from the charged surface, and the ferroelectric polarization attract charges on top Cu surface to bottom Cu electrode. The ferroelectric polarization and attracted charges on Cu electrode are saturated when the gap reaches maximum point (Fig. 6a(iii)). The attracted charges are transferred back to the top Cu electrode as the top Cu and PTFE get close and make contact again, but the induced ferroelectric polarization in BT remains (Fig. 6a(iv)–(v)). The remaining ferroelectric polarization can enhance triboelectric charge generation on PTFE surface during contact electrification. Figure 6b shows the output performance of TENG that charge density is 142 μC/m^2^ at atmosphere condition and increases up to 1003 μC/m^2^ at high vacuum (10^−6^ torr). The gaseous breakdown voltage increases as the gas pressure decreases (Fig. 6b(vi)), so it is possible to enhance output charge density using BT layer at high vacuum. Due to the high charge density, open-circuit voltage and short-circuit current increased up to 180 V and 570 mA/m^2^, respectively. The maximum output power was shown at a load resistance of 10 MΩ and enhanced up to 50 W/m^2^ with BT layer and high vacuum.

The application of TENG with BT layer in electronic devices exhibited in Fig. 6c. The supercapacitor is charged by closing K1 and opening K2 in an equivalent circuit, described in Fig. 6c(i). The charged voltage in supercapacitor is 21.49 mV for a charging time of 10 s in atmosphere, but the voltage increases up to 80.36 mV in high vacuum conditions, as shown in Fig. 6c(ii). The high output TENG in high vacuum shows rising voltage of supercapacitor even while the watch and humidity-temperature meter are working (Fig. 6c(iii)). On the other hand, when the TENG works in atmosphere, the supercapacitor hardly charged (Fig. 6c(iv)) or the charged voltage decreased (Fig. 6c(vi)). The high output TENG also shows the ability to light 32 light-emitting diodes (LEDs) when working in high vacuum (Fig. 6c(vii)), but only two LEDs were lit in atmosphere (Fig. 6c(viii) and (ix)). The introduction of ferroelectric layer in TENG shows the enhanced charge density beyond the limit of conventional materials, particularly in high vacuum where breakdown voltage increases.

### Pyroelectric energy harvesting

#### Pyroelectric effect

The pyroelectric effect is the polarization in certain crystals which have polar crystal structures by change in temperature [44, 45]. The atoms in crystal shift by change in temperature, and it results in variation of electric field and voltage across the material (primary pyroelectric effect). Therefore the pyroelectric coefficient (p) is expressed as follows:

3 $$ {\text{p }} = \left( {\frac{{\partial P_{s} }}{\partial T}} \right)_{E,\sigma } $$

where *P*_*s*_ is the spontaneous polarization, *T* the temperature, *E* the electric field, and *σ* the elastic stress [45]. As we can see in Eq. (3), pyroelectricity occur when the crystal is heated or cooled. The short circuit current by pyroelectric effect is described:

4 $$ I = \frac{{{\text{d}}Q}}{{{\text{d}}t}} = {\text{p}}A\frac{{{\text{d}}T}}{{{\text{d}}t}} $$

where *Q* is pyroelectric charge, *t* is time, p is pyroelectric coefficient, A is surface area of material [45]. In terms of crystal classes, all pyroelectric materials have piezoelectricity as well. Therefore, thermal expansion by temperature charge induce mechanical stress resulting in piezoelectric polarization, which is called secondary pyroelectric effect. The total pyroelectric coefficient is the sum of the primary and secondary pyroelectric effect.

For energy harvesting from thermal energy, the thermoelectric effect has been used [46–48]. However, it requires a spatial gradient in temperature, so it is not applicable when the temperature of material varies [49]. Therefore, when there is time-dependent variation of temperature, pyroelectric energy generator (PEG) can be used for energy harvesting [4, 50–55].

#### Pyroelectric energy harvesting from hot/cold water

Heat energy is one of the most prevalent types of wasted energy, and there have been many researches to harvest the heat energy. Conventionally, thermoelectric technology has been exploited to convert heat energy to electric energy, but temperature gradient should be maintained for thermoelectric effect and conversion efficiency is still low [49]. Therefore, pyroelectric energy generator (PEG) was invented [50], but output power of inorganic pyroelectric material-based PEGs is still low (voltage of 22 V, and current of 170 nA) due to the low pyroelectric coefficient of PZT (− 80 nC/cm^2^K) [53]. However, polymeric ferroelectric material, PVDF is promising material for PEG because of its high pyroelectric coefficient (200 μC/cm^2^K) [56], and its flexibility enables it to be applied for flexible and stretchable PEG [54].

**Fig. 7:**
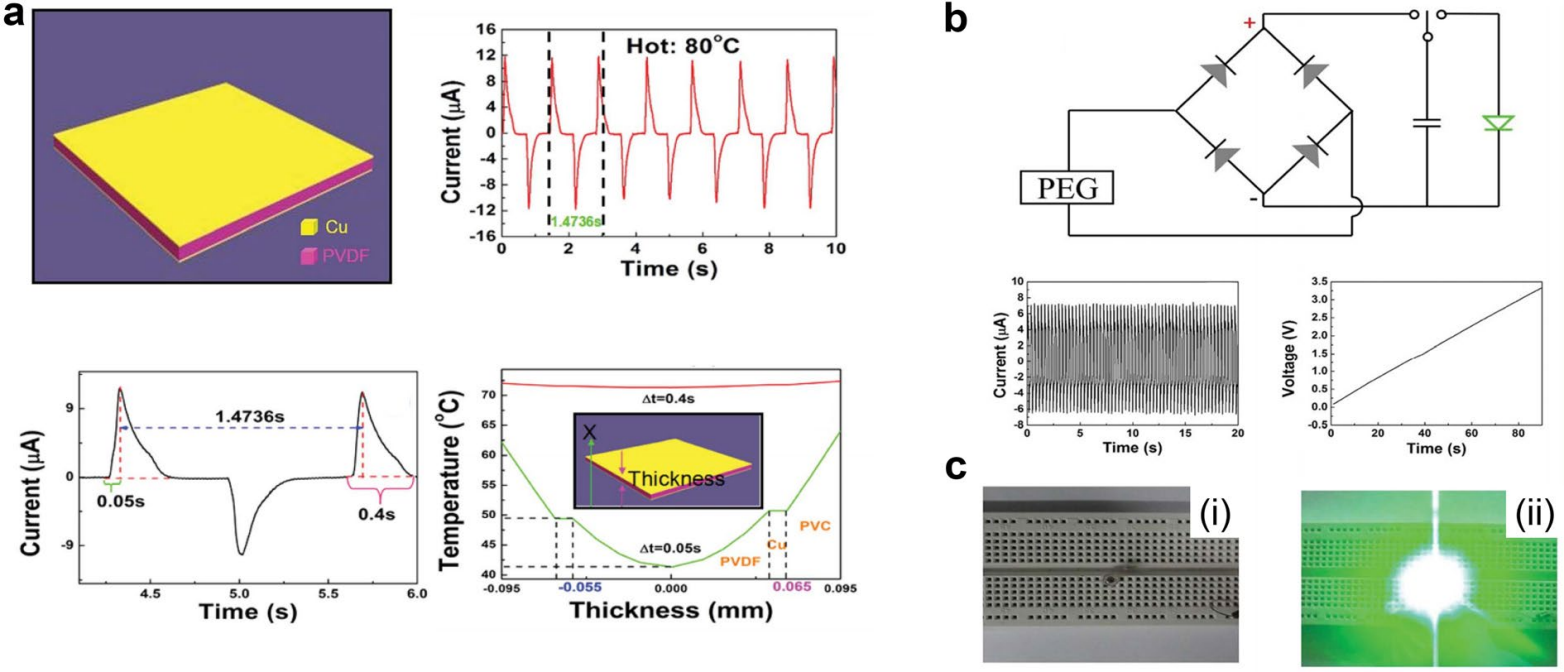
**a** Schematic structure of P(VDF-TrFE) based PNG and output current by contacting hot and cold water alternatively. **b** Equivalent circuit for charging system and capacitor charging behavior. **c** Switching on LED by PNG (Reproduced from [55] with copyright permission from 2014 Royal Society of Chemistry)

Leng et al. [55] developed flexible PEG using PVDF film as shown in Fig. 7a. Thin Cu layers are deposited on both top and bottom surfaces as electrode, and the device contacts cold (0 °C) and hot (40, 60, 80 °C) water alternatively. The maximum output current by contact with hot (80 °C) and cold water is 12 μA. However, the PVDF has a thickness of 110 μm and is covered by a 10 μm Cu electrode and 30 μm PVC, so there is temperature gradation in the device. At the initial state its temperature is the same as room temperature (20 °C), but when it is soaked in the hot water (80 °C) the temperature of PVDF film is not uniform across the thickness. The surface temperature increases up to 50 °C, and then to around 72 °C. According to Eq. (4), the calculated current at middle and surface of PVDF is 11.42 μA and 14.58 μA respectively, which shows good agreement with experimental result. In addition, the electric energy from PEG was stored in capacitor (100 μF). Figure 7b illustrates equivalent circuit for charging capacitor and charging behavior. The charging voltage–time curve shows voltage of capacitor charged up to 3.3 V in 90 s, and the LED was switched on by charged capacitor as shown in Fig. 7c. In this report, PVDF based PEG shows a high output performance for energy harvesting.

#### Highly stretchable piezoelectric-pyroelectric coupled energy harvester

Recently, the electronic devices are required to be flexible and stretchable as well for application of wearable electronics [57–61]. As mentioned before, ferroelectric polymer P(VDF-TrFE) has a lot of advantageous features for such an application. Especially stretchability is one of the most unique properties of P(VDF-TrFE) among the ferroelectric materials. Besides, dual properties of pyroelectricity and piezoelectricity in P(VDF-TrFE) can realize high output energy harvester by hybridization. The stretchable hybrid energy harvester is successfully realized through the introduction of micro-line pattern structure and combining piezoelectric and pyroelectric effect [54]. However, the piezoelectric and pyroelectric effect output was produced by each independent energy source.

**Fig. 8:**
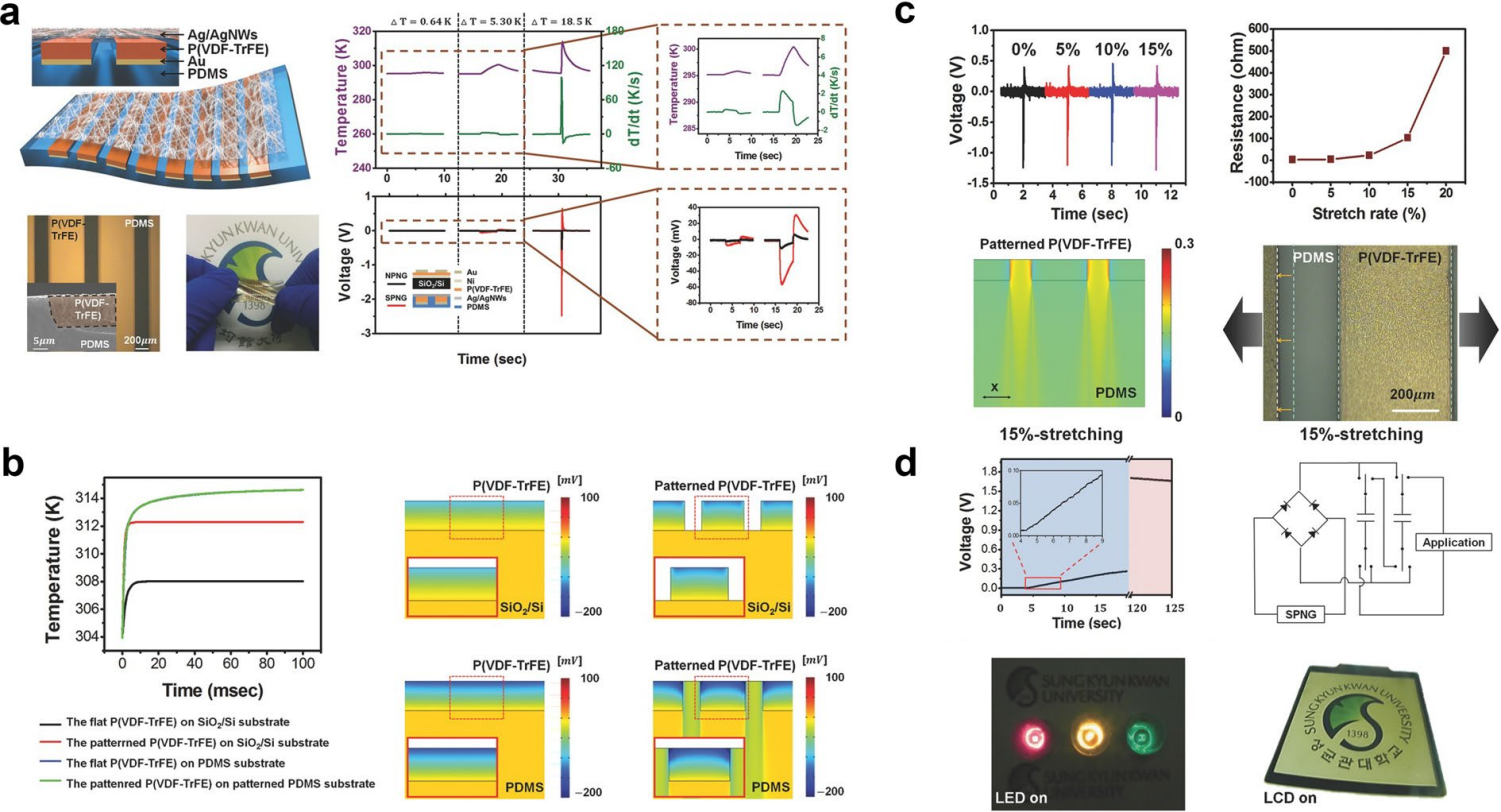
**a** Structure of micro-patterned P(VDF-TrFE) based SPNG and output current comparison. **b** Temperature dynamics of each devices and piezoelectric potential by thermal expansion. **c** Mechanical stability under stretching. **d** Demonstration of SPNG for charging capacitor and switching on LEDs and LCD (Reproduced from [4] with copyright permission from 2015 John Wiley & Sons)

Lee et al. [4] introduced novel design of stretchable pyroelectric nanogenerator (SPNG) by introducing micro-patterned PDMS for coupling piezoelectric and pyroelectric effect using different thermal expansion as shown in Fig. 8a. P(VDF-TrFE) and PDMS have different thermal expansion coefficients 122 × 10^−6^ K^−1^ [62], and 310 × 10^−6^ K^−1^ [63] respectively, so compressive strain is applied to P(VDF-TrFE) resulting in piezoelectric effect. Output voltage of SPNG and normal pyroelectric nanogenerator (NPNG) which is composed of flat P(VDF-TrFE) on Ni/SiO_2_/Si substrate was compared at each temperature change rate. The SPNG shows output voltages of 8 mV to 2.48 V at temperature variations of 0.64 K to 18.5 K whereas the NPNG shows 2 mV to 0.54 V at the same temperature variations. Temperature distributions of micro-patterned P(VDF-TrFE) and flat P(VDF-TrFE) on PDMS substrate and SiO_2_/Si substrate are compared. Figure 8b shows average temperature of each device as a function of time and piezoelectric potential by thermal expansion. The calculated average temperature is highest in case of the micro-patterned P(VDF-TrFE) because of its larger area on which heat is applied and low thermal conductivity of PDMS (0.15 Wm^−1^K^−1^ [63]) than SiO_2_ (1.5 Wm^−1^K^−1^ [64]) and Si (129 Wm^−1^K^−1^ [64]). Due to difference in thermal expansion coefficients of PDMS and P(VDF-TrFE), compressive stress is applied to patterned P(VDF-TrFE) from the neighboring PDMS resulting in enhancement of piezoelectric potential. The micro-patterned structure shows mechanical stability under stretching as described in Fig. 8c. Both pyroelectric potential of the SPNG and its top electrode resistance is almost maintained at almost same value. Young’s modulus of PDMS (0.36 GPa) is much lower than P(VDF-TrFE) (3 GPa), so when the device is stretched tensile strain is more concentrated at PDMS as demonstrated with FEM simulation. Optical microscopy images also show good agreement with the simulation results. Figure 8d shows the voltage of the capacitor which is charged up to 1.7 V within 210 s using high output SPNG. Finally, the operation of LEDs and liquid crystal display (LCD) by SPNG connected to integrated circuit with charging system was demonstrated.

### Photovoltaic energy harvesting

#### Ferroelectric effect in photovoltaic cell

Solar energy is one of the most abundant energy sources in the earth, and photovoltaic cells using solar energy are currently the most prevalent energy harvesting technology. In order to increase the output power of photovoltaic cells, controlling electronic properties like energy band structure or junction is essential [65, 66]. Ferroelectric materials have switchable spontaneous polarization once electric field is applied. When the ferroelectric layer is introduced in photovoltaic cell, the polarization induces internal electric field resulting in aid separation of excited carriers. Therefore, there have been reports about ferroelectric-inserted photovoltaic devices. Moreover, it is found that recently developed photovoltaic material, organic halide perovskite has ferroelectric polarization.

#### Ferroelectric coupling on energy-band structure

**Fig. 9:**
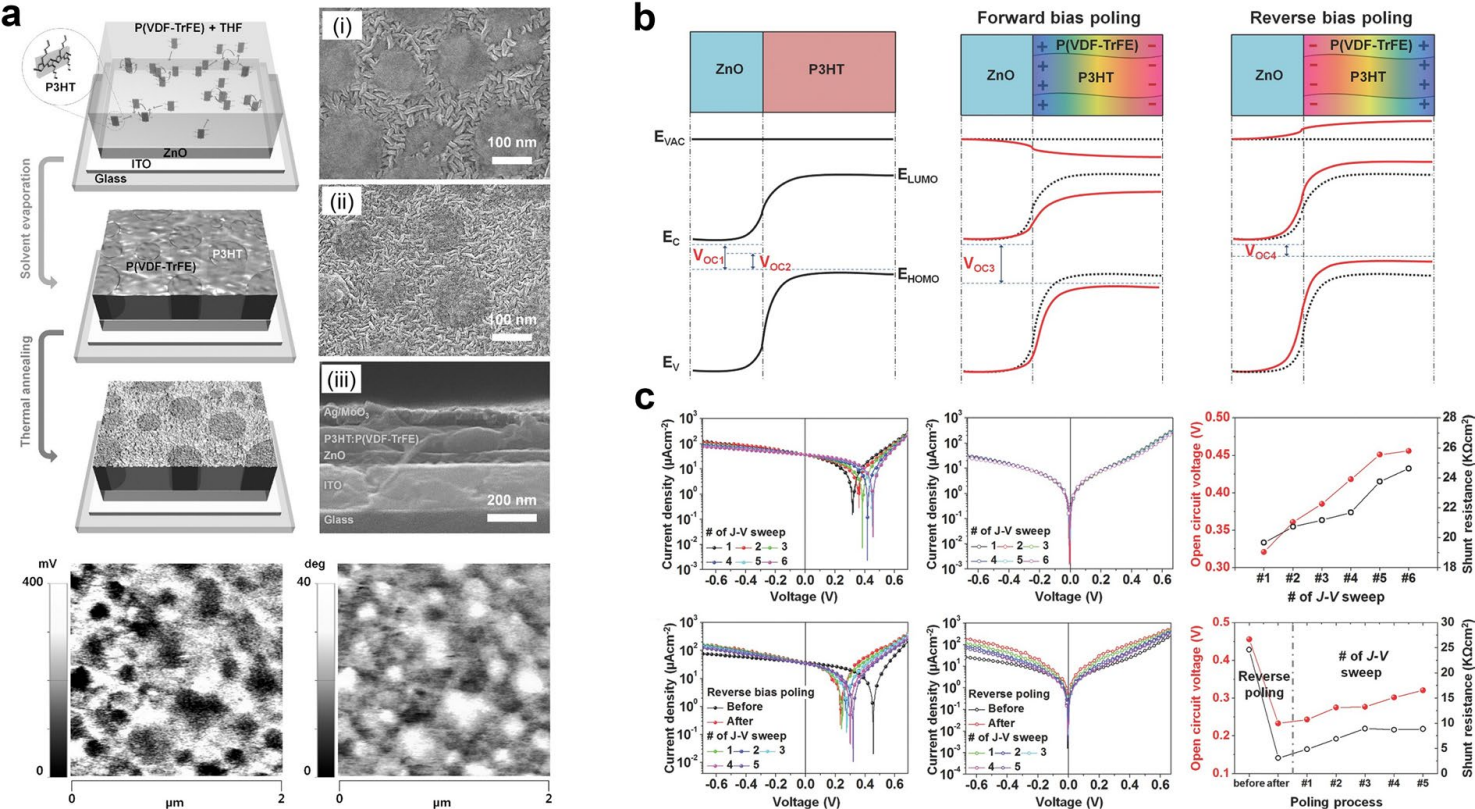
**a** Phase separation of P(VDF-TrFE) and P3HT. **b** Energy band structure of P(VDF-TrFE):P3HT/ZnO junction modified by ferroelectric polarization. **c** Ferroelectric polarization-affected V_OC_ and J_SC_ (Reproduced from [75] with copyright permission from 2014 John Wiley & Sons)

There are several reports of controlling energy level of junction in photovoltaic cell by inserting ferroelectric layer [67–74]. These methods introduced novel technique to increase output current and voltage but increased contact resistance as well resulting in reduction of output power [70]. Shin et al. [75] designed novel structure of P(VDF-TrFE) self-organized nanomatrix to increase output performance without increasing contact resistance. Figure 9a shows that phase separation of P(VDF-TrFE) and P3HT was formed by preferential interaction and thermal annealing of the P(VDF-TrFE):P3HT blend solution. Ferroelectric polarization is found at the P(VDF-TrFE) region and phase separation is observed by PFM measurement, similar to the morphology image. The energy band structures of photovoltaic cells with P3HT/ZnO (PZ-device) and P(VDF-TrFE):P3HT/ZnO (PPZ-device) with ferroelectric polarization are illustrated in Fig. 9b. N-type ZnO and p-type polymer P3HT form p–n junction and V_OC_ is determined by difference between electron quasi-Fermi energy of ZnO and hole quasi-Fermi energy of P3HT (V_OC1_) [76–79]. However, the recombination of excitons resulted from defects in sol–gel based ZnO [80, 81], reducing the difference of energy level and V_OC_ (V_OC2_) [82, 83]. In the case of PPZ-device, polarization in forward bias poled P(VDF-TrFE) increases energy level difference (V_OC3_). On the other hand, polarization in reverse biased P(VDF-TrFE) decreases energy level difference (V_OC4_). The J–V sweep curve of PPZ-device in Fig. 9c shows poling dependence of V_OC_ and J_SC_. As number of J–V sweeps increases V_OC_ increases from 0.318 to 0.456 V and J_SC_ increase as well. However, when the device is reversely poled, the V_OC_ decreases from 0.456 V to 0.233 V, but V_OC_ is recovered as number of J–V sweep increases. In this report, it is found that the ferroelectric layer can modify and enhance the output performance of a photovoltaic cell.

#### Ferroelectric behavior in halide perovskite solar cell

Recently, one of the most promising materials for photovoltaic cells is halide perovskite [84, 85]. The power conversion efficiency (PCE) of perovskite photovoltaic cell that fabricated with two-step sequential deposition and vapor evaporation method achieved 15% in 2013 [86, 87]. After that, much higher PCEs of perovskite photovoltaic cell have been reported [3, 88]. The halide perovskite material, CH_3_NH_3_PBI_3_ (MAPbI_3_) has unique properties such as ambipolar self-doping property [89], high permittivity [90], I–V hysteresis [91], and slow dynamics [92]. It is expected that MAPbI_3_ has ferroelectricity [93], but this is still ambiguous as of now.

**Fig. 10:**
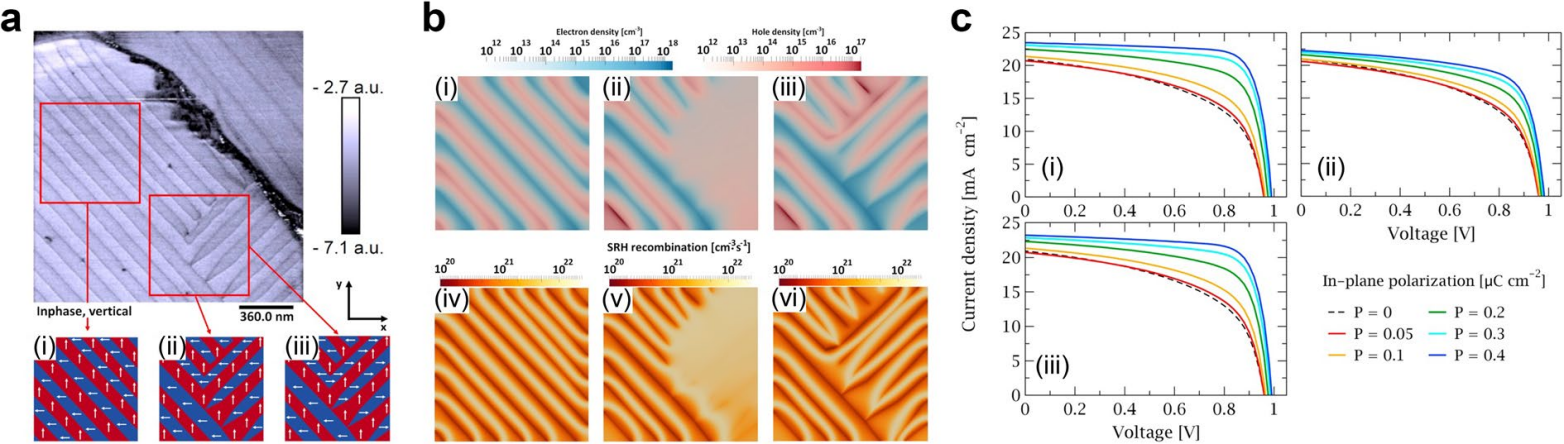
**a** PFM image of MAPbI_3_(Cl) domain and polarization orientation. **b** Simulations of charge carrier densities and SRH recombination at corresponding region of a. **c** Simulation of J–V characteristic with in-plane polarization (Reproduced from [95] with copyright permission from © 2018 Elsevier Ltd.)

Röhm et al. [94] found and measured the ferroelectric domain in MAPbI_3_(Cl) using PFM. From the PFM measurement result, carrier behavior and J–V characteristic were computed with each ferroelectric polarization alignment case [95]. Figure 10a shows the PFM measurement image with stripe pattern and three assumed ferroelectric polarization directions: (i) alternative head-to-head and tail-to-tail pattern, (ii) domain with perpendicular corner but without change in polarization direction resulting in head-to-tail orientation, (iii) same domain shape as (ii) but polarization direction is reversed at corner. The charge carrier densities and Shockley–Read–Hall (SRH) recombination were computed using drift–diffusion model for three cases of ferroelectric polarization directions as described in Fig. 10b. The carriers are attracted and accumulated along the domain interfaces (Fig. 10b(i)–(iii)), and the recombination rate decreases at the domain interfaces as well (Fig. 10b(iv)–(vi)). However, the head-to-tail oriented region in Fig. 10b(ii) does not show separation, and a high recombination rate is observed. Further calculation of J–V characteristics for three cases by light harvesting is exhibited in Fig. 10c. As in-plane polarization increases from 0 to 0.4 μC/cm^2^, V_OC_, J_SC_, and fill factor (FF) increases because charge separation become more efficient whereas recombination declines. The V_OC_ increased by 30 mV and FF increased from 52 to 77% at polarization of 0.4 μC/cm^2^ for alternative head-to-head and tail-to-tail pattern (Fig. 10c(i), (iii)). Therefore, power conversion efficiency (PCD) calculation shows enhancement from 11 to 18.5%. However, the enhancement by ferroelectric polarization is not noticeable in head-to-tail orientation case (Fig. 10c(ii)). As described in Fig. 10b, carrier recombination rate in head-to-tail oriented region is high so charge separation occurs hardly, resulting in weak dependence on in-plane polarization density. In conclusion, from PFM measurement of ferroelectric polarization in MAPbI_3_(Cl) and the orientation assumption, charge separation and recombination were analyzed by simulation. The simulation results showed orientation dependence and the results affected J–V characteristics depending on in-plane ferroelectric polarization. Therefore, it can be considered that well-ordered ferroelectric polarization can enhance the output power of MAPbI_3_(Cl) solar cell.

## Summary and future prospect

The unique properties of the ferroelectric effect, particularly its spontaneous, switchable, and permanent polarization have attracted many researchers to develop many application devices, and energy harvesting technologies have exploited the unique properties of ferroelectric material. Energy harvesters convert various energy sources to electrical energy. Ferroelectric polarization can have an important role to increase output power of energy harvesters by enhancing internal potential. Strong ferroelectric polarization produces high piezoelectric potential and surface potential. For mechanical stability and robustness in PENG and TENG, the oxide ferroelectric materials were deposited in thin film or imbedded in polymer matrix. Using oxide ferroelectric powder-polymer composite and ferroelectric polymer P(VDF-TrFE), very highly stable PENG and stretchable PENG were developed. In the case of TENG, controlling the surface potential is crucial. The ferroelectric polarization modified and attracted more charges, resulting in higher output power. With high output TENG, it was demonstrated that electronic devices such as smart watch and humidity-temperature meter can be driven and charged simultaneously. In addition, ferroelectric polymer P(VDF-TrFE) which has pyroelectricity was micro-patterned in order to develop stretchable PEG, and hybrid pyroelectric effect and piezoelectric effect for high output. Finally, it was found that the energy level at junction in photovoltaic cell can be adjusted to increase V_OC_ and J_SC_. Recently, ferroelectric polarization was discovered in MAPbI_3_ which is considered as a promising photovoltaic material and studied.

Nowadays, the importance of energy harvesting technologies has become larger due to the prevalence of the mobile electronics and the fact that their power consumption is very high. However, present energy storage technology cannot cover the power consumption needs, so the output power of energy harvester must be improved. There are several factors to determine output power of energy harvesters, but the development of a proper material is a key factor. The introduction of ferroelectric material will give way for improvement in designing material system in energy harvester and realize alternative powering system in near future.
